# Sequential and Simultaneous Interactions of Plant Allelochemical Flavone, Bt Toxin Vip3A, and Insecticide Emamectin Benzoate in *Spodoptera frugiperda*

**DOI:** 10.3390/insects14090736

**Published:** 2023-08-31

**Authors:** Kaiyuan Huang, Haibo He, Shan Wang, Min Zhang, Xuewei Chen, Zhongyuan Deng, Xinzhi Ni, Xianchun Li

**Affiliations:** 1 School of Agricultural Sciences, Zhengzhou University, Zhengzhou 450001, Chinahhb167113q@gmail.com (H.H.); sunnywang0507@163.com (S.W.); zhangmin753@gmail.com (M.Z.); chen_xw@zzu.edu.cn (X.C.); 2 School of Life Sciences, Zhengzhou University, Zhengzhou 450001, China; 3 USDA-ARS, Crop Genetics and Breeding Research Unit, University of Georgia-Tifton Campus, Tifton, GA 31793, USA; xinzhi.ni@usda.gov; 4 Department of Entomology and BIO5 Institute, University of Arizona, Tucson, AZ 85721, USA

**Keywords:** allelochemical, Bt toxin, chemical insecticide, combined toxicity, induced toxicity, synergistic interactions

## Abstract

**Simple Summary:**

The widespread cultivation of genetically engineered crops producing not only toxic proteins from the bacterium *Bacillus thringiensis* (Bt) but also plant defensive compounds known as allelochemicals, combined with occasional use of insecticides, is the major tactic to manage some economically important pests. Better understanding of the toxicological interactions of the three types of toxins is needed to rationally deploy them to protect crops from pests. The aim of this study is to examine the sequential and simultaneous interactions of the allelochemical flavone, Bt toxin Vip3A, and insecticide emamectin benzoate in the fall armyworm (*Spodoptera frugiperda*), a worldwide target pest of Bt crops. Bioassays of *S. frugiperda* neonates revealed that all interactions of the three toxins, except for 1-day pre-exposure to a sublethal dose (LC_5_) of flavone followed by 6-day simultaneous exposure to flavone LC_5_ + emamectin benzoate LC_50_, are synergistic or additive. The results suggest that the combined use of the three toxins is basically a great strategy to manage *S. frugiperda*.

**Abstract:**

Target pests of genetically engineered crops producing both defensive allelochemicals and *Bacillus thuringiensis* (Bt) toxins often sequentially or simultaneously uptake allelochemicals, Bt toxins, and/or insecticides. How the three types of toxins interact to kill pests remains underexplored. Here we investigated the interactions of Bt toxin Vip3A, plant allelochemical flavone, and insecticide emamectin benzoate in *Spodoptera frugiperda*. Simultaneous administration of flavone LC_25_ + Vip3A LC_25_, emamectin benzoate LC_25_ + Vip3A LC_25_, and flavone LC_15_ + emamectin benzoate LC_15_ + Vip3A LC_15_ but not flavone LC_25_ + emamectin LC_25_ yielded a mortality significantly higher than their expected additive mortality (EAM). One-day pre-exposure to one toxin at LC_5_ followed by six-day exposure to the same toxin at LC_5_ plus another toxin at LC_50_ showed that the mortality of flavone LC_5_ + Vip3A LC_50_, emamectin benzoate LC_5_ + Vip3A LC_50_, and Vip3A LC_5_ + emamectin benzoate LC_50_ were significantly higher than their EAM, while that of flavone LC_5_ + emamectin benzoate LC_50_ was significantly lower than their EAM. No significant difference existed among the mortalities of Vip3A LC_5_ + flavone LC_50_, emamectin benzoate LC_5_ + flavone LC_50_, and their EAMs. The results suggest that the interactions of the three toxins are largely synergistic (inductive) or additive, depending on their combinations and doses.

## 1. Introduction

Plants and herbivorous insects have antagonistically co-evolved for over 400 million years, whereby plants acquire various morphological and chemical defenses to protect themselves from herbivores, and insects disarm plant defenses for food, survival, and reproduction [1,2,3,4,5]. Among plant chemical defenses are various toxic plant secondary metabolites (i.e., allelochemicals, such as tannins, cyanide, glycosides, alkaloids, terpenoids, saponins, flavonoids, furanocoumarins, indoles, and phytoecdysteroids), nonprotein or unusual amino acids (e.g., canavanine and 3-hydroxyproline), plant defense proteins/enzymes [e.g., lectins, proteinase inhibitors (PIs), peroxidases (POD), polyphenol oxidases (PPO)], and volatile organic compounds (VOCs) [1,4,5,6,7,8,9,10]. Direct use and/or augmentation of the natural defenses of crops, especially breeding and planting insect-resistant varieties that produce more anti-herbivore allelochemicals, are one of the major tactics for integrated pest management [7].

The advancement in biotechnology from the 1980s to 1990s has enabled the introduction of insecticidal toxin or protein genes from other organisms into the genomes of crops, making *in planta* production of pest-resistant toxins of foreign source possible [11,12,13,14,15,16]. Commercialization of genetically engineered crops expressing insecticidal proteins from *Bacillus thuringiesis* (Bt) in 1996 marked the beginning of a new paradigm in pest management—from labor-intensive and pollution-inevitable toxin sprays to *in planta* self-production of toxins [17]. Transgenic Bt crops can provide a safe and highly effective control of major insect pests such as the European corn borer, southwestern corn borer, tobacco budworm, cotton bollworm, pink bollworm, and fall armyworm while decreasing use of conventional insecticides, boosting biological control, and enhancing yields [18,19,20,21,22]. These economic, health, and environmental benefits have led to rapid global adoption of transgenic Bt crops. The area planted globally to transgenic Bt crops increased from 1 million hectares (ha) in 1996 to 109 million ha in 2019, which accounted for >53% of the global cultivated area of genetically modified crops [23]. The Bt crops planted by millions of farmers in 27 nations in 2019 include corn, cotton, soybean, sugarcane, and eggplant [23].

Other than expressing the introduced Bt toxins such as Cry1Ac, Cry2Ab, and Vip3A, Bt crops, like all plants, also produce their own insect-resistant allelochemicals. This makes target pests less likely to survive on Bt crops because they have a double “green prevention and control” shield for these pests. Nonetheless, supplementary insecticide sprays may be necessary to address the surviving insects when high dose of Bt toxins cannot be reached [24,25], Bt toxin concentrations decline in late season [26], or practical resistance of pests to Bt toxins occurs [27]. Consequently, pests feeding on Bt crops are inevitable to ingest low or high doses of Bt toxin proteins sequentially or simultaneously, naturally occurring plant defense allelochemicals/proteins, and/or insecticides, depending on the growth stage of the plants being attacked [26].

Whether the three types of toxins synergistically or antagonistically interact with each other when simultaneously ingested and induce or inhibit each other’s toxicity under sequential ingestion remains underexplored. Sachs et al. [28] showed that pyramiding Bt toxin Cry1Ab and terpenoid in cotton provided a higher yet additive level of resistance to *Heliothis virescens* larvae than Cry1Ab or terpenoid alone. Other reported additive cases include tannic acid + Cry1Ac against *Helicoverpa armigera* [29], leptin glycoalkaloid *+* Cry3A against *Leptinotarsa decemlineata* [30], and gossypol plus Cry1Ac + Cry2Ab against *Spodoptera littoralis* [31]. Among the reported synergistic cases are azadirachtin + *Bacillus thuringiensis* Berliner sub sp. kurstaki against *H. armigera* [32], maize insect resistance cysteine protease (Mir1-CP) + Cry2A against *Helicoverpa zea*, *H. virescens*, *Spodoptera frugiperda,* and *Diatraea grandiosella* [33], gossypol + Cry1Ac against a resistant strain of *H. zea* [34], jasmonic acid-induced resistance plus Cry1Ac or Cry1Ac + Cry2Ab against *S. frugiperda* [35], and flavone + Cry1Ac against *H. armigera* [36]. The antagonistic cases are tannis + Cry1Ac against *H. armigera* [37], quercetin + Cry1Ac against *H. armigera* [38], and Bt + trichlorfon against *Plutella xylostella* [39]. So far, there has been only one sequential ingestion case study, which showed that pre-exposure to Cry1Ac significantly induced flavone’s toxicity against *H. armigera*, whereas pre-exposure to flavone did not induce or inhibit Cry1A’s toxicity against the same pest species [36].

In this study, we used *S. frugiperda*, an invasive polyphagous target pest of Bt crops, to address the questions of whether sequential or simultaneous ingestion of flavone, a representative of the ubiquitous flavonoids [40,41,42]; Vip3A, one of the most widely deployed Bt toxins in Bt transgenic crops [23,43]; and emamectin benzoate, an effective insecticide for controlling this pest [44,45,46,47], induce and/or synergize each other’s toxicity. Our results reveal that simultaneous ingestion of lethal doses of the three toxins and any two of the three toxins caused a synergistic interaction for flavone + Vip3A, emamectin benzoate + Vip3A, and flavone + emamectin benzoate + Vip3A but an additive interaction for flavone + emamectin benzoate. When any two of the three toxins were sequentially ingested, emamectin benzoate induced Vip3A’s toxicity, flavone induced Vip3A’s toxicity but inhibited emamectin benzoate’s toxicity, and neither of Vip3A and emamectin benzoate affected flavone’s toxicity.

## 2. Materials and Methods

### 2.1. Insect Sources

A laboratory strain of *S. frugiperda* was used in the current study. The strain was received as a gift from Dr. Shaohua Gu at the China Agricultural University in September 2020 and, thus, named CAU strain hereafter. Based on their description [48], the CAU strain was established with *S. frugiperda* larvae collected from a maize field in Yilang city (24.76° N, 103.15° E), Yunnan Province, China, in June 2019. Pupae used in the experiment were soaked in 5% formaldehyde for 5 min, washed with water and placed into a clean cage to wait for adult emergence. Inside cages, a 1 oz plastic cup with a cotton ball soaked with 10% sugar water solution was provided for adult supplementary feeding, as well as a piece of cheesecloth for female oviposition. Pieces of cheesecloth used in the experiment had been sterilized by soaking in 5% formaldehyde solution for 30 s, washing with tap water, and drying at room temperature. The egg masses on the collected pieces of cheesecloth were put in plastic cups until they hatched out. Within 6 h, the neonate larvae were kept at 26 ± 1 °C and 60 ± 10% relative humidity (RH) with a 16: 8 h (L/D) photoperiod on a semisynthetic diet containing wheat germ [49].

### 2.2. Preparation of Toxin-Containing Diet

Vip3A protoxin, flavone (reagent grade), and emamectin benzoate (abbreviated as emamectin hereafter) were procured from Beijing Honoster Biotechnology Company (Beijing, China), Shanghai Aladdin Biochemical Technology Company (Shanghai, China), and China Agricultural University, respectively. Acetone, pH 7.4 phosphate (PBS) buffer, and triton X-100 were obtained from Luoyang Haohua Chemical Reagent Co., LTD (Luoyang, Henan, China), Sangon Biotech Company (Shanghai, China), and Solarbio Company (Beijing, China), respectively.

The toxin diets were prepared by diet incorporation for flavone, diet overlay for Vip3A, emamectin, or their mixture, and a combination of diet incorporation and overlay for mixtures involving flavone plus Vip3A, emamectin, or both Vip3A and emamectin. As the diets cooled to approximately 47 °C, 300 μL of flavone solution or acetone (solvent control for flavone) was added to 30 mL diets, vortexed vigorously, and apportioned 0.75 mL to each well of 128-well bioassay trays (C-D International, Pitman, NJ, USA). The diets with overlayed toxins were prepared by dispensing 0.75 mL diets in each well of bioassay trays first. After the diets coagulated, we covered the diets of each well with 60 μL of a given concentration of Vip3A protoxin, emamectin, 0.25 × pH 7.4 PBS buffer (Vip3A protoxin solvent), or acetone (emamectin solvent). We then put the bioassay trays on an orbital shaker rotating at 70 r/m for 30 min to entail formation of a uniform layer of Vip3A, emamectin, or the two solvents on the surface of the diets. Preparation of the diets with both incorporated and overlayed toxins began by vigorously vortexing 30 mL diets incorporated with 300 μL of a given concentration of flavone or acetone, followed by dispensing the diet mixtures to wells (0.75 mL diets/well) of bioassay trays, overlaying 60 μL of a given concentration of Vip3A protein, emamectin, 0.25 × pH 7.4 PBS buffer (Vip3A protoxin solvent), or acetone (emamectin solvent) on the surface of the solidified flavone or acetone-incorporated diets, and finally rotating the bioassay trays on an orbital shaker at 70 r/m for 30 min. The double overlay (overlay+ overlay) diets were made by allotting 0.75 mL of diet in each well of bioassay trays, followed by coating the coagulated diets with 60 μL of a given concentration of Vip3A protoxin or 0.25 × pH 7.4 PBS buffer (Vip3A protoxin solvent), overlaying 60 μL of a given concentration of emamectin or acetone (emamectin solvent), and orbiting the trays on an orbital shaker at 70 r/m for 30 min.

### 2.3. Bioassay

To determine the toxicity of Vip3A protoxin and emamectin, a total of 48 neonate larvae (3 repeats of 16 insects) per treatment (different concentrations and control) of *S. frugiperda* hatched within 6 h were transferred onto the diet surface of each well (1 neonate per well) containing 0.75 mL diet that overlaid with 0.25 × pH 7.4 PBS buffer (0.05% triton X-100), acetone (with 0.05% triton X-100), different concentrations of Vip3A protoxin, or different concentrations of emamectin. A similar procedure was followed to measure the toxicity of flavone except that flavone- and acetone-incorporated (control) diets were used. The bioassay trays with neonate larvae were maintained at 26 ± 1 °C, 60 ± 10% R.H., and a photoperiod of 16: 8 h (L/D). The numbers of dead and live larvae in each of the 1st to the 4th instars were recorded after 7 d, respectively. The sums of dead and retarded (1st instar) larvae were used to calculate larval mortalities for each control and concentration of the three toxins.

The yielded lethal dose–probit regression lines (LD–P line) of the three toxins were used to calculate the LC_5_, LC_15_, LC_25_, and LC_50_ doses of each toxin. To assess the combined toxicities of two-toxin (flavone LC_25_ + Vip3A LC_25_, Vip3A LC_25_ + emamectin LC_25_, and flavone LC_25_ + emamectin LC_25_) and three-toxin mixtures (flavone LC_15_ + Vip3A LC_15_ + emamectin LC_15_), 48 neonate larvae (3 repeats of 16 insects each) were transferred to wells (1 larva/well) of the bioassay trays containing the corresponding solvent control diets, single toxin diets (flavone: LC_15_ = 197 µg/mL, LC_25_ = 300.08 µg/mL; Vip3A: LC_15_ = 0.03 μg/cm, LC_25_ = 0.06 μg/cm; or emamectin: LC_15_ = 0.06 ng/cm, LC_25_ = 0.09 ng/cm), two-toxin mixture diets, and three-toxin mixture diets, respectively.

To examine if one day pre-administration of a sublethal dose (≤LC_10_) of flavone induces or inhibits the toxicity of Vip3A or emamectin, a total of 192 neonates were exposed to the acetone- or flavone (LC_5_ = 89.5 µg/mL diet)-incorporated diets, respectively. After 24 h, the 192 larvae on the diets incorporated with LC_5_ dose of flavone were divided into 4 quarters of 48 larvae each (3 replicates of 16 larvae each) and then the 4 quarters were transferred to the diets incorporated with flavone LC_5_ and covered with 0.25 × pH 7.4 PBS buffer (flavone only treatment for flavone LC_5_ + Vip3A LC_50_), the diets mixed with flavone LC_5_ and coated with 0.01% acetone (flavone only treatment for flavone LC_5_ + emamectin LC_50_), the diets contained flavone LC_5_ and covered with Vip3A LC_50_ (0.1μg/cm) (flavone LC_5_ + Vip3A LC_50_), and the diets contained flavone LC_5_ and covered with emamectin LC_50_ (0.12 ng/cm) (flavone LC_5_ + emamectin LC_50_), respectively. Likewise, we transferred 4 quarters of the neonate larvae from the acetone-incorporated diet after 24 h to the diets containing acetone and covered with pH 7.4 0.25 × PBS (Vip3A control), the diets supplemented with acetone and coated with acetone (emamectin control), the diets mixed with acetone and covered with Vip3A LC_50_ (Vip3A only treatment), and the diets contained acetone and coated with emamectin LC_50_ (emamectin only treatment), respectively. In the same manner, bioassays of *S. frugiperda* neonates with Vip3A LC_5_ (0.008 μg/cm), emamectin LC_5_ (0.039 ng/cm), flavone LC_50_ (689.5 µg/mL), Vip3A LC_50_ (0.1 μg/cm), emamectin LC_50_ (0.12 ng/cm), Vip3A LC_5_ + flavone LC_50_, Vip3A LC_5_ + emamectin LC_50_, emamectin LC_5_ + Vip3A LC_50_, emamectin LC_5_ + flavone LC_50_, and the corresponding solvent controls were carried out to uncover whether one day earlier exposure to Vip3A LC_5_ induces/inhibits the toxicity of flavone/emamectin as well as whether one day earlier exposure to emamectin LC_5_ induces/inhibits the toxicity of Vip3A/flavone.

All the aforementioned combined and induced toxicity bioassay treatments and controls were maintained at 26 ± 1 °C, 60 ± 10% R.H., with a 16: 8 h (L/D) photoperiod. The numbers of dead and live larvae in each instar (1st to the 4th) were recorded after 7 d, respectively. We used the sum of the dead and retarded (1st instar) larvae for calculation of larval mortality.

### 2.4. Data Analysis

The adjusted mortalities of all concentrations and treatments were calculated with Abbott’s formula [50]. Probit analysis was performed to estimate the dose-response lines, LC_50_, LC_25_, and LC_5_ doses of flavone, Vip3A, and emamectin against *S. frugiperda* neonate larvae using the SPSS software (SPSS, 1998). The differences among the adjusted mortalities of the single toxin alone treatments and sequential or simultaneous combination treatments were compared using one-way ANOVA, followed by the Tukey’s honestly significant difference (HSD) test at *p* < 0.05 in GraphPad Prism, https://www.graphpad.com/ (accessed on 20 June 2023) (GraphPad Software Inc., La Jolla, CA, USA). We used the Chi-squared test and co-toxicity factor [51] assay to ascertain the nature of interaction [i.e., synergistic (inductive), additive, or antagonistic (inhibitive)] among flavone, Vip3A, and emamectin. If the Chi-squared test revealed that the observed adjusted mortality of a consecutive or concomitant mixture was <, =, or > the expected additive mortality (EAM = sum of adjusted mortalities of the corresponding single toxins) of two or three toxins, then a conclusion of antagonistic (or inhibitive), additive, or synergistic (inductive) interaction between the toxins could be drawn. The co-toxicity factor was computed using the following equation:

$$Co-toxocity\;factor=\frac{Observed\;mortality\;(\%)-EAM\;(\%)}{EAM\;(\%)}\times 100.$$

The co-toxicity factor within the range of >+20, −20 to +20, and <−20 represented antagonistic (inhibitive), additive, and synergistic (inductive) interaction, respectively [51].

## 3. Results

### 3.1. Toxicity of Vip3A, Flavone, and Emamectin against S. frugiperda Neonate Larvae

The obtained lethal dose–probit regression lines (LD–P line), three lethal doses (LC_15_, LC_25_, and LC_50_) and one sublethal (LC_5_) dose of flavone, Vip3A, and emamectin against the neonate larvae of the CAU strain of *S. frugiperda* were summarized in Table 1. Based on their LC_50_ values, emamectin was about 1150-fold more toxic than Vip3A, and Vip3A was over 3000-fold more potent than flavone (Table 1). Given that the LC_50_ of the CAU strain for Vip3A [161 ng/cm^2^ (95% FL: 90–370)] was about the same with that of a Vip3A-susceptible strain [156.496 ng/cm^2^ (95%FL: 87.15–217.96)] [52], the CAU strain is susceptible to Vip3A.

### 3.2. Toxic Synergy among Vip3A, Emamectin Benzoate, and Flavone against S. frugiperda Neonate Larvae

**Figure 1:**
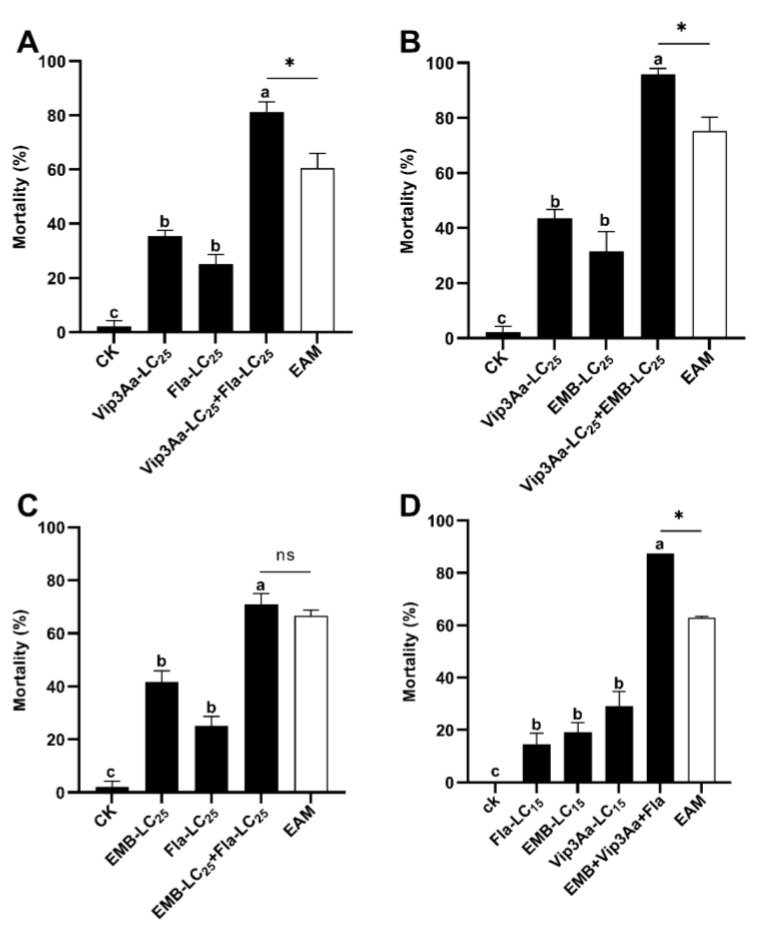
Combined toxicity of Vip3A + flavone (**A**), Vip3A + emamectin (**B**), flavone + emamectin (**C**), and Vip3A + flavone + emamectin (**D**) against *Spodoptera frugiperda* neonates. Unconverted average mortalities ± SE (standard error) are presented. Mortality data were arcsine converted prior to statistical analysis. Within each small graph, mean mortalities followed by the same letter are not significantly different at *p* > 0.05 (Tukey’s HSD test). Significant difference is depicted by an asterisk (*) between the observed mortality and expected additive mortality (EAM) of each mixture at *p* < 0.05 (Chi-square test). Fla = flavone, EMB = emamectin benzoate, ns = No significance.

Simultaneous exposure of *S. frugiperda* neonates to the calculated LC_25_ doses of either two of the three toxins (Vip3A, emamectin and flavone) (Table 1) were conducted to determine the types of their interactions (additive, synergistic, or antagonistic). The corrected mortalities for exposure to the calculated LC_25_ doses of Vip3A (0.06 μg/cm) and flavone (300.08 µg/mL) were 35.42% and 25%, respectively (Table 2, Figure 1A). When *S. frugiperda* neonates were simultaneously treated with LC_25_ doses of Vip3A and flavone, the corrected mortality was 81.25%, yielding a co-toxicity factor of >20 (Table 1), the cutoff value for a significant synergistic interaction between toxins. Consistent with the co-toxicity factor value (34.48 > 20), the observed corrected mortality of the flavone-Vip3A combination was significantly higher than that of the two single toxin treatments and the expected additive mortality (EAM = 35.42% + 25% = 60.42%) of the two toxins (Figure 1A) (Tukey’s HSD and Chi-square tests).

When *S. frugiperda* neonates concurrently ingested the calculated LC_25_ doses of Vip3A (0.06 μg/cm) and *emamectin* (0.09 ng/cm), we observed an adjusted mortality of 95.83%, which was significantly higher than that of each toxin and their EAM (43.61% + 31.65% = 75.26%) (Tukey’s HSD and Chi-square tests) and resulted in a co-toxicity factor of 27.34 (>20, the criterium for synergistic interaction) (Table 2, Figure 1B). By contrast, simultaneous exposure to the LC_25_ doses of flavone (300.08 µg/mL) and emamectin (0.09 ng/cm) caused an adjusted mortality of 70.83% and a co-toxicity factor of 6.5, belonging to the scope of additive interactions (Table 2). Tukey’s HSD and Chi-squared tests uncovered that the adjusted mortality of the flavone/emamectin mixture was significantly higher than that of each toxin alone but was not different from their EAM (25.0% + 41.67% = 66.67%) (Table 2, Figure 1C).

We also tested the combined toxicity of Vip3A, flavone, and emamectin to *S. frugiperda* neonates, using a ratio of LC_15_: LC_15_: LC_15_. The three-toxin combination produced an adjusted mortality of 87.5% and a co-toxicity factor of 40.0 (>20), suggesting a synergistic toxicological interplay among the three toxins (Table 2). The corrected mortality of the three-toxin mixture was significantly higher than that of flavone (197.0 µg/g diets, 14.58%), Vip3A (0,03 μg/cm^2^, 29.17%), emamectin (0.06 ng/cm^2^, 19.17%), and their EAM (14.58% + 29.17% + 19.17% = 62.92%) (Table 2, Figure 1D) (Tukey’s HSD and Chi-squared tests).

### 3.3. Asymmetrical Toxicity Induction between Flavone and Vip3A against S. frugiperda Neonates

**Figure 2:**
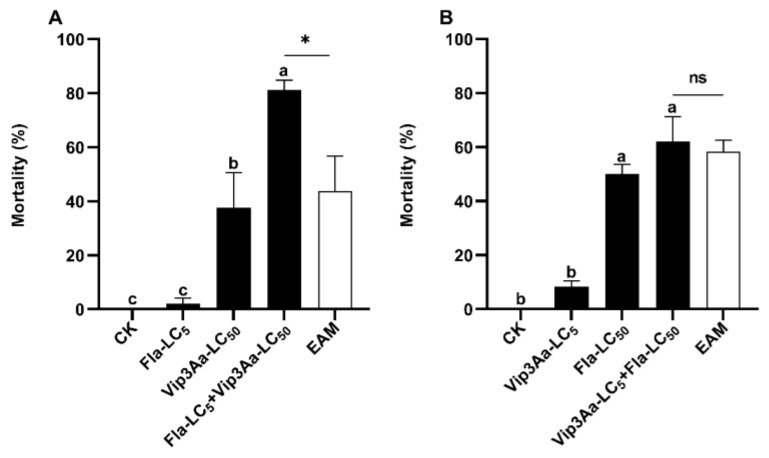
Induced toxicity of flavone LC_5_ + Vip3A LC_50_ (**A**) and Vip3A LC_5_ + flavone LC_50_ (**B**) against *Spodoptera frugiperda* neonates. Untransformed average mortality ± SE are presented. Mortality data were arcsine converted prior to statistical analysis. Within each small graph, mean mortalities followed by the same letter are not significantly different at *p* > 0.05 (Tukey’s HSD tests). Significant difference is depicted by an asterisk (*) between the observed mortality and the expected additive mortality (EAM) of each mixture at *p* < 0.05 (Chi-squared test). Fla = flavone, ns = No significance.

One day earlier exposure of *S. frugiperda* neonates to the LC_5_ sublethal dose of flavone (89.5 µg/g diets) followed by 6-day exposure to the LC_5_ sublethal dose of flavone and the LC_50_ dose of Vip3A (0.1 μg/cm) (flavone LC_5_ + Vip3A LC_50_) effected a corrected mortality of 81.25% and a co-toxicity factor of 85.71 (Table 3). Tukey’s HSD and Chi-squared tests uncovered that the corrected mortality caused by flavone LC_5_ + Vip3A LC_50_ was significantly greater than that of the larvae feeding on the diets with the LC_5_ sublethal dose of flavone for 7 d (flavone LC_5_) and of the larvae feeding for 1 d on the diets with acetone (flavone solvent) and then for 6 d on the diets with LC_50_ dose of Vip3A (Vip3A LC_50_) and the EAM of flavone LC_5_ and Vip3A LC_50_ (6.25% + 37.5% = 43.75%) (Table 3, Figure 2A).

By contrast, one day earlier exposure of *S. frugiperda* neonates to the LC_5_ sublethal concentration of Vip3A (0.008 μg/cm^2^) and then 6-day exposure to the LC_5_ sublethal concentration of Vip3A plus the LC_50_ dosage of flavone (689.6 µg/g diets) (Vip3A LC_5_ + flavone LC_50_) provoked an adjusted mortality of 62.08% and a co-toxicity factor of 6.43 (Table 3), which fell within the range (−20 to 20) of additive interactions [50]. Consistent with this inference, the correct mortality elicited by Vip3A LC_5_ + flavone LC_50_ was not greater than that of the larvae exposed to 0.25 × PBS buffer (Vip3A solvent) for 1 d then to the LC_50_ dose of flavone for 6 d (flavone LC_50_) and the EAM of Vip3A LC_5_ + flavone LC_50_ (Tukey’s HSD and Chi-square tests) (Table 3 and Figure 2B).

### 3.4. Asymmetrical Toxicity Induction between Vip3a and Emamectin Benzoate against S. frugiperda Larvae

**Figure 3:**
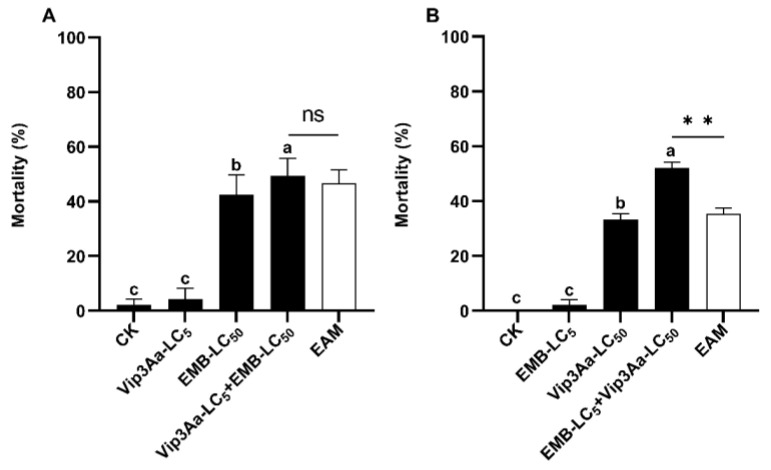
Induced toxicity of Vip3A LC_5_ + emamectin LC_50_ (**A**) and emamectin LC_5_ +Vip3A LC_50_ (**B**) against *Spodopterra frugiperda* neonates. Untransformed average mortality ± SE are presented. Mortality values were arcsine converted prior to analysis. Within each small graph, mean mortalities followed by the same letter are not significantly different at *p* > 0.05 (Tukey’s HSD tests). Extremely significant difference is indicated by two asterisks (**) between the observed mortality and the expected additive mortality (EAM) of each mixture at *p* < 0.01 (Chi-squared test). EMB = emamectin benzoate, ns = No Significance.

The corrected mortality for *S. frugiperda* neonates exposed to LC_5_ sublethal dose of Vip3A (0.008 μg/cm^2^) for 1 d followed by 6 d of LC_5_ sublethal dose of Vip3A plus LC_50_ dose of emamectin (0.12 ng/cm^2^) (Vip3A LC_5_ + emamectin LC_50_) was 49.4%, yielding a co-toxicity factor of 5.86 (Table 3). This mortality was significantly higher than that of the larvae fed for 1 d on the diets overlaid with 0.25 × PBS buffer (Vip3A solvent) and then for 6 d on the diets covered with LC_50_ dosage of emamectin (emamectin LC_50_) but not different from the expected additive mortality (EAM = 46.67%) of Vip3A LC_5_ (4.17%) and emamectin LC_50_ (42.5%) (Tukey’s HSD and Chi-square tests) (Table 3, Figure 3A).

When *S. frugiperda* neonates were first exposed to the LC_5_ sublethal concentration of emamectin (0.039 ng/cm^2^) and then to the LC_5_ sublethal concentration of emamectin plus LC_50_ dose of Vip3A (0.1 ug/cm) (emamectin LC_5_ + Vip3A LC_50_), we observed a corrected mortality of 52.08% and a co-toxicity factor of 47.06 (Table 3), belonging to the range of induction. Tukey’s HSD and Chi-squared tests revealed that the adjusted mortality caused by emamectin LC_5_ + Vip3A LC_50_ was notably higher than that of Vip3A LC_50_ (33.33%) and the EAM (35.42%) of Vip3A LC_50_ and emamectin LC_5_ (2.08%) (Table 3, Figure 3B).

### 3.5. Asymmetrical Toxicity Inhibition between Flavone and Emamectin Benzoate against S. frugiperda Neonates

**Figure 4:**
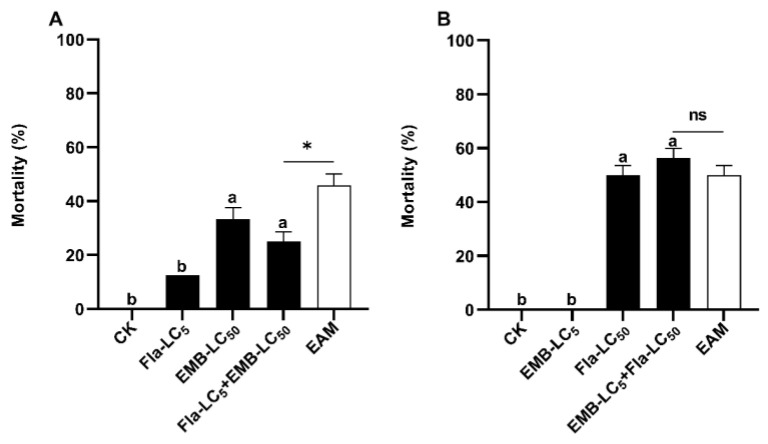
Induced toxicity of flavone LC_5_ + emamectin LC_50_ (**A**) and emamectin LC_5_ + flavone LC_50_ (**B**) against *Spodoptera frugiperda* neonates. Untransformed average mortality ± SE are presented. Mortality data were arcsine converted prior to analysis. Within each small graph, mean mortalities followed by the same letter are not significantly different at *p* > 0.05 (Tukey’s HSD tests). Significant difference is depicted by one asterisk (*) between the observed mortality and the expected additive mortality (EAM) of each mixture at *p* < 0.05 (Chi-squared test). Fla = flavone, EMB = emamectin benzoate, ns = No Significance.

Seven-day exposure to LC_5_ sublethal dose (89.5 µg/g diets) of flavone (flavone LC_5_) and 1-d exposure to acetone (flavone solvent) plus subsequent 6-d exposure to LC_50_ dose (0.12 ng/cm^2^) of emamectin (emamectin LC_50_) resulted in an adjusted death rate of 12.5% and 33.33% (Table 3), respectively. Sequential exposure to the two toxins, i.e., 1-d exposure to flavone LC_5_ followed by 6-d exposure to flavone LC_5_ plus emamectin LC_50_ (flavone LC_5_ + emamectin LC_50_), yielded a corrected mortality of 25.0% and co-toxicity factor of −45.45, far below the critical value (−20) for inhibitive interaction (Table 3). In agreement with this inference, the corrected mortality of flavone LC_5_ + emamectin LC_50_ was significantly lower than that of the EAM (45.83%) of flavone LC_5_ and emamectin LC_50_ (Table 3, Figure 4A) (Tukey’s HSD and Chi-squared tests).

When the order of sequential exposure to flavone and emamectin was reversed, the corrected mortality (56.25%) of emamectin LC_5_ + flavone LC_50_ was not significantly different from that (50.0%) of flavone LC_50_ (689.6 µg/g diets) and the EAM (0% + 50% = 50%) of flavone LC_50_ and emamectin LC_5_ (0.039 ng/cm^2^) (Table 3, Figure 4B) (Tukey’s HSD and Chi-squared tests). The co-toxicity factor was 12.5, which fell within the range of additive interaction (−20 to <20) (Table 3, Figure 4B).

## 4. Discussion

*S. frugiperda* and other target pests of Bt-transgenic crops may concurrently or sequentially encounter Bt toxins, anti-herbivore allelochemicals and/or insecticides, depending on the spatiotemporal expression patterns of Bt toxins [53,54] and plant defense allelochemicals [55,56,57] as well as the timing of insecticide sprays. Binary and ternary mixtures of flavone, Vip3A, and emamectin devised to imitate concurrent consumption of the three types of poisons exhibited a toxicological synergy for flavone + Vip3A, emamectin + Vip3A, and flavone + emamectin + Vip3A but an additive interaction for flavone + emamectin (Table 2 and Figure 1). In theory, synergy can arise only if the mixed poisons reciprocally elevate each other’s toxicity or at least one of the mixed poisons boosts the potency of the other one or two poisons. Our induced toxicity experiments simulated the sequential intake of any two of the three toxins detected a significant induction effect of one-day earlier feeding of a sublethal concentration (LC_5_) of flavone and emamectin on the toxicity of Vip3A LC_50_ but did not find an induction effect of one-day earlier ingestion of Vip3A LC_5_ on the toxicity of flavone LC_50_ and emamectin LC_50_ (Table 3 and Figure 2 and Figure 3). On the contrary, consuming flavone LC_5_ one day in advance inhibited the toxicity of emamectin LC_50_, but consuming emamectin LC_5_ one day in advance did not affect the toxicity of flavone LC_50_ (Table 3 and Figure 4). The asymmetrical induction of the poisonousness hints that the synergy found in the mixtures of flavone + Vip3A, emamectin + Vip3A, and Flavone + emammectin + Vip3A (Table 2 and Figure 1) likely resulted from the elevation of Vip3A toxicity by flavone, emamectin, or both, rather than reciprocally strengthening each other’s toxicity. The fact that the co-toxicity factor values of the binary mixtures of flavone + Vip3A and emamectin + Vip3A were about half of those of the sequential mixtures of flavone LC_5_ + Vip3A LC_50_ and emamectin LC_5_ + Vip3A LC_50_ (Table 3) supports this speculation.

There are three possible routes by which flavone and/or emamectin can enhance the toxicity of Vip3A. Flavone and/or emamectin may synergistically upregulate the expression of the receptors of Vip3A, such as the putative Vip3Aa-binding ribosomal protein S2 [58] and/or the Vip3A-activated apoptosis pathway genes [59]. They may also upregulate the protease genes responsible for the activation of Vip3A, such as trypsin [60]. The third approach is to improve the accessibility of Vip3A to its receptor protein(s) by promoting Vip3A’s entrance into its receptor sites and/or decreasing its degradation, accumulation, and/or evacuation. In agreement with the three possible routes, flavone is known to up- and down-regulate 295 and 125 genes, respectively, in *Spodopera litura* [61], a sister species of *S. frugiperda*, and 38 and 10 genes, respectively, in *H. armigera* [62]. By the same token, emamectin can up- and down-regulate 599 and 1658 genes, respectively, in the predatory beetle *Paederus fuscipes* [63], and 1495–2784 and 1622–2351 genes, respectively, in *Spodoptera exigua*, depending on the dose of emamectin [64]. Additional experiments are required to illuminate how flavone and emamectin induce and elevate the potency of Vip3A to *S. frugiperda*.

On the other hand, the sequential flavone inhibition of emamectin toxicity failed to decide the type or nature of concurrent interactions between emamectin and flavone since an additive but not antagonistic interaction was observed for the binary mixture of flavone + emamectin (Table 2 and Figure 1). This suggests that the nature of simultaneous interactions of two or more toxins may not be always explained by their sequential interactions, especially when asymmetrical inhibition occurs. Our finding of asymmetrical inhibition of emamectin toxicity by flavone is consistent with the lack of cross-resistance to the plant allelochemical 2-tridecanone in the insecticide (fenvalerate)-resistant Colorado potato beetle [65] as well as the unidirectional (asymmetrical) cross-resistance to several organophosphate pesticides of the triterpenoid cucurbitacin-C-selected two-spotted spider mite [66] and to the pyrethroid insecticide a-cypermethrin of the plant allelochemical xanthotoxin-exposed *H. zea* survivors and their offspring [67]. Along the same line, pre-exposure to flavone effectively enhanced detoxification enzyme activities and larval tolerance to multiple synthetic insecticides in *Spodoptera litura* by turning on the ROS-CncC-mediating xenobiotic metabolism pathway [61]. Pre-ingestion of the flavonoids catechin, myricetin kaempferol, quercetin, and rutin markedly enhanced P450 activity and resistance to flupyradifurone and thiamethoxam in *Bemisia tabaci* [68]. And, preexposure to visnagin, DIMBOA (2,4-Dihydroxy-7-methoxy-1,4-benzoxazin-3-one), coumarin, and flavone significantly decreased larval susceptibility of *H. armigera* to methomyl via upregulation of P450s including *CYP6B2*, *CYP6B6*, and *CYP6B7* [69]. Such unidirectional sequential inhibition and unidirectional cross-resistance between insecticides and anti-herbivore allelochemicals are probably prompted by the absence of behavioral adjustment to the repellent and/or antifeedant influences of plant allelochemicals in insecticide pre-exposed individuals or survivors, rather than by the lack of metabolic adaptation [67]. After all, insecticides are also capable of inducing metabolic enzymes [64,67], and at least some of the induced or selected detoxification enzymes are capable of metabolizing both insecticides and allelochemicals [67].

The resistance of pests to Bt-transgenic crops and insecticides is the major challenge for pest management. Developing new Bt crops that can dramatically enhance the manufacture of anti-herbivore allelochemicals/proteins is one possible strategy to address this challenge [31,70], exposing target pests to the redundant and synergistic killing effect of the two or even three (when spraying insecticides) types of toxins. The basic requirements for anti-herbivore allelochemicals/proteins to be co-expressed with Bt insecticidal proteins in new Bt crops include the following: (1) Additive or synergistic interaction with the corresponding Bt toxins and even with the commonly used insecticides if possible; (2) Negative or no cross-resistance with the corresponding Bt toxins and even with insecticides if possible; (3) Natural presence in major crops. The allelochemical flavone apparently meets the third requirement as it is naturally present in a wide range of plants including corn, cotton, and soybean [40,41,42]. The data reported here demonstrate that flavone also meets the first requirement as it synergistically interacted with Vip3a and additively with emamectin against *S. frigiperda*. Moreover, flavone is known to toxicologically synergize with Cry1Ac [36] and Cry2Ab (unpublish data) against *H. armigera* and with Cry1Ab against *S. frugiperda* (unpublish data) (He H., personal communication). Given the popularity of Cry1Ab, Cry1Ac, Cry105, Cry2Ab, and Vip3a in Bt crops (https://www.texasinsects.org/bt-corn-trait-table.html; accessed on 23 June 2023), flavone would be a suitable allelochemical to be stacked with these Bt toxins to manage pests if further cross-resistance studies confirm that it also meets the second requirement.

## Figures and Tables

**Table 1:** Toxicity of Vip3Aa, flavone, and emamectin benzoate against *Spodoptera frugiperda* neonate larvae.

Toxins	N ^a^	LD-P Line	LC_5_ (95% FL ^b^)	LC_25_ (95% FL ^b^)	LC_50_ (95% FL ^b^)	*R* ^2 c^
Vip3Aa	285	Y = 1.97X + 1.56	24 ^d^ (1–50)	73 (19–130)	161 (90–370)	0.89
Flavone	275	Y = 3.05X − 8.27	147.95 ^e^ (27.0–244.6)	307.5 (142.1–348.1)	511.1 (354.6–801.8)	0.91
Emamectin benzoate	285	Y = 3.50X + 2.99	0.047 ^d^ (0.03–0.06)	0.09 (0.074–0.1)	0.14 (0.12–0.16)	0.96

^a^—Number of neonates tested. ^b^—95% Fiducial limits. ^c^—Correlation coefficient. ^d^—ng/cm2 for Vip3Aa and Emamectin benzoate. ^e^—ug/g Diets for flavone.

**Table 2:** Co-toxicity factors of concurrent exposure of *Spodoptera frugiperda* neonates to emamectin benzoate, Vip3Aa, and flavone.

Exposure to		Observed Mortality (%)	EAM * (%)	Co-Toxicity Factor	Interaction
Concurrent Vip3Aa_LC_25_ + Fla_LC_25_	Vip3Aa	35.42 ± 2.08 ^a^			
	Fla ^b^	25.00 ± 3.61			
	Vip3Aa + Fla	81.25 ± 3.61	60.42	+20 < 34.48	Synergism
Concurrent Vip3Aa_LC_25_ + EMB_LC_25_	Vip3Aa	43.61 ± 3.06			
	EMB ^c^	31.65 ± 7.01			
	Vip3Aa + EMB	95.83 ± 2.08	75.26	+20 < 27.34	Synergism
Concurrent Fla_LC_25_ + EMB_LC_25_	Fla	25.00 ± 3.61			
	EMB	41.67 ± 4.17			
	Vip3Aa + EMB	70.83 ± 4.17	66.67	+20 > 6.25	Addition
Concurrent Fla_LC_15_ + EMB_LC_15_ + Vip3Aa_LC_15_	Fla	14.58 ± 4.17			
	EMB	19.17 ± 3.63			
	Vip3Aa	29.17 ± 5.51			
	Fla + EMB + Vip3Aa	87.50 ± 0.00	62.92	+20 < 40.00	Synergism

^a^—Standard error of the means. ^b^—Flavone. ^c^—Emamectin benzoate. * EAM= expected additive mortality.

**Table 3:** Co-toxicity factors of in sequence exposure of *Spodoptera frugiperda neonates* to emamectin benzoate, Vip3Aa, and flavone.

Exposure to		Observed Mortality (%)	EAM * (%)	Co-Toxicity Factor	Interaction
Sequential Vip3Aa_LC_5_ + Fla_LC_50_	Vip3Aa	8.33 ± 2.08 ^a^			
	Fla	50.00 ± 3.61			
	Vip3Aa + Fla ^b^	62.08 ± 9.14	58.33	−20 < 6.43 < +20	Addition
Sequential Fla_LC_5_ + Vip3Aa_LC_50_	Fla	6.25 ± 0.00			
	Vip3Aa	37.50 ± 13.01			
	Fla + Vip3Aa	81.25 ± 3.61	43.75	+20 < 85.71	Induction
Sequential Fla_LC_5_ + EMB_LC_50_	Fla	12.5 ± 0.00			
	EMB ^c^	33.33 ± 4.17			
	Fla + EMB	25.00 ± 3.61	45.83	−45.45 < −20	Inhibition
Sequential EMB_LC_5_ + Fla_LC_50_	EMB	0.00 ± 0.00			
	Fla	50.00 ± 3.61			
	EMB + Fla	56.25 ± 3.61	50.00	−20 < 12.50 < +20	Addition
Sequential Vip3Aa_LC_5_ + EMB_LC_50_	Vip3Aa	4.17 ± 4.17			
	EMB	42.50 ± 7.32			
	Vip3Aa + EMB	49.40 ± 6.37	46.67	−20 < 5.86 < +20	Addition
Sequential EMB_LC_5_ + Vip3Aa_LC_50_	EMB	2.08 ± 2.08			
	Vip3Aa	33.33 ± 2.08			
	EMB + Vip3Aa	52.08 ± 2.08	35.42	+20 < 47.06	Induction

^a^—Standard error of the means; ^b^—Flavone; ^c^—Emamectin benzoate; * EAM = expected additive mortality.

## Data Availability

The data presented in this study are available within in the article.
